# Supplementary material for: Identification of photoperiod-induced specific miRNAs in the adrenal glands of Sunite sheep (Ovis aries)
Source: Front Vet Sci. 2022 Jul 22;9:888207. doi: 10.3389/fvets.2022.888207 (PMC9354845; doi:10.3389/fvets.2022.888207)
Supplement: Supplementary file 1 [file Data_Sheet_1.ZIP › Supplementary file/miRDeep2 file.zip!/miRDeep2 file/Novel_Structure/SP42A3/Novel_658.pdf]

Provisional ID	:	NC_019484.2_32648
Score total	:	1.9
Score for star read(s)	:	-1.3
Score for read counts	:	0
Score for mfe	:	1.6
Score for randfold	:	1.6
Score for cons. seed	:	
Total read count	:	341
Mature read count	:	341
Loop read count	:	0
Star read count	:	0

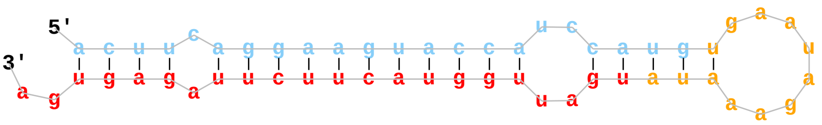

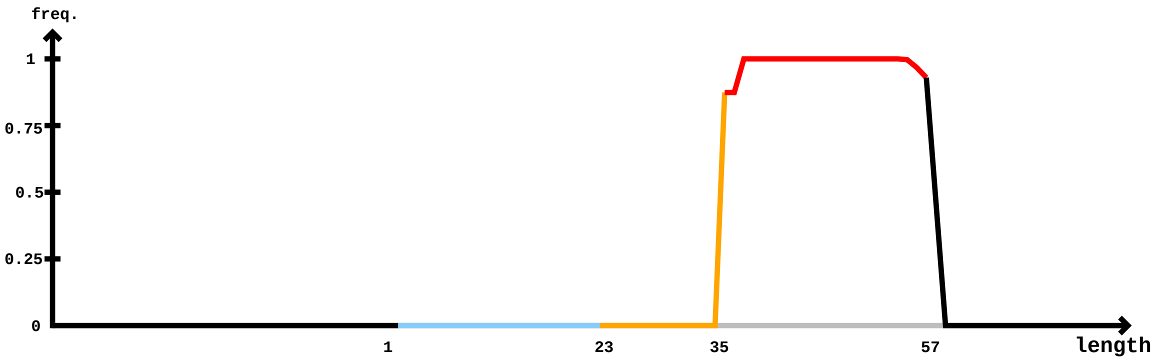

Star Mature

```
5'- ucaccacauuucacuaugccgcgugcaguauugcacuucaggaaguaccauccaugugaauagaaauaugauugguacuucuuagagugaaguaaugcauagcaaauaca   -3'    exp
    ................(((...(((((.((((.(((((.(((((((((((..(((((........)))))..))))))))))).))))).)))).))))).)))......    reads      mm         sample
    ....................................................................ugauugguacuucuuagag.......................    1          0          seq
    ....................................................................ugauugguacuucuuagagu......................    10         0          seq
    ....................................................................ugauugguacuucuuagagug.....................    12         0          seq
    ....................................................................ugauugguacuucuuagaguga....................    239        0          seq
    ....................................................................ugauugguacuucuuagagugaa...................    36         0          seq
    ......................................................................auugguacuucuuagagug.....................    1          0          seq
    ......................................................................auugguacuucuuagaguga....................    25         0          seq
    ......................................................................auugguacuucuuagagugaa...................    17         0          seq
```
